# Parasites, Drugs and Captivity: *Blastocystis*-Microbiome Associations in Captive Water Voles

**DOI:** 10.3390/biology10060457

**Published:** 2021-05-22

**Authors:** Emma L. Betts, Sumaiya Hoque, Lucy Torbe, Jessica R. Bailey, Hazel Ryan, Karen Toller, Vicki Breakell, Angus I. Carpenter, Alex Diana, Eleni Matechou, Eleni Gentekaki, Anastasios D. Tsaousis

**Affiliations:** 1 Laboratory of Molecular and Evolutionary Parasitology, RAPID Group, School of Biosciences, University of Kent, Canterbury CT2 7NJ, UK; elb48@kent.ac.uk (E.L.B.); sh986@kent.ac.uk (S.H.); lucyrtorbe@gmail.com (L.T.); bailey2j09@virginmedia.com (J.R.B.); 2 Wildwood Trust, Herne Common, Herne Bay CT6 7LQ, UK; hazel@wildwoodtrust.org (H.R.); karen.toller@wildwoodtrust.org (K.T.); vicki@wildwoodtrust.org (V.B.); 3 School of Animal, Rural and Environmental Sciences, Brackenhurst Campus, Nottingham Trent University, Nottinghamshire NG1 4FQ, UK; carpenter.angus@gmail.com; 4 School of Mathematics, Statistics and Actuarial Science, University of Kent, Canterbury CT2 7NJ, UK; A.Diana@kent.ac.uk (A.D.); E.Matechou@kent.ac.uk (E.M.); 5 School of Science and Human Gut Microbiome for Health Research Unit, Mae Fah Luang University, Chiang Rai 57100, Thailand

**Keywords:** *Blastocystis*, captivity, microbiome, polyparasitism, water voles

## Abstract

**Simple Summary:**

The last decade has seen a large increase into research on the microbiome and its roles in health and disease. The majority of this work has focused primarily on the bacterial component of the microbiome. However, there is evidence to suggest that microbial eukaryotes colonising the gastrointestinal tract may have roles in the shaping and structuring of the microbiota and are thus likely to influence disease outcomes and host health. The aim of this study was to investigate the questionable pathogen *Blastocystis* and expand the number of studies on non-primate hosts, which address its associations with bacterial communities in the gut. Herein we examined the bacterial gut microbiota of *Blastocystis* positive and negative water voles. Results demonstrate no association of *Blastocystis*, bacterial richness and community composition. Nonetheless, the abundance of some taxa was affected in *Blastocystis* positive samples. The lack of significant shifts in community abundance between *Blastocystis* carriers and non-carriers indicates that this microbe may not be having a profound impact on bacterial communities in the gut of these animals.

**Abstract:**

(1) Background: *Blastocystis* is a microbial eukaryote inhabiting the gastrointestinal tract of a broad range of animals including humans. Several studies have shown that the organism is associated with specific microbial profiles and bacterial taxa that have been deemed beneficial to intestinal and overall health. Nonetheless, these studies are focused almost exclusively on humans, while there is no similar information on other animals. (2) Methods: Using a combination of conventional PCR, cloning and sequencing, we investigated presence of *Blastocystis* along with *Giardia* and *Cryptosporidium* in 16 captive water voles sampled twice from a wildlife park. We also characterised their bacterial gut communities. (3) Results: Overall, alpha and beta diversities between water voles with and without *Blastocystis* did not differ significantly. Differences were noted only on individual taxa with *Treponema* and *Kineothrix* being significantly reduced in *Blastocystis* positive water voles. Grouping according to antiprotozoal treatment and presence of other protists did not reveal any differences in the bacterial community composition either. (4) Conclusion: Unlike human investigations, *Blastocystis* does not seem to be associated with specific gut microbial profiles in water voles.

## 1. Introduction

The gastrointestinal tract is a dynamic and varied ecosystem made up of trillions of bacteria, viruses, protozoa, fungi and archaea that co-evolved with the host [1]. As a result, mutually beneficial interactions have developed over a prolonged period of time. Recently, there has been an explosion of studies focusing on the microbiome and its role in host health and disease. Nonetheless, such studies are largely anthropocentric and focus mainly on bacterial microbiota [2,3,4,5,6]. Microbiome-based research of non-human vertebrates mainly encompasses livestock [7,8,9], companion animals [10,11] and other notable species, such as those at risk of extinction [12,13]. A common objective of animal studies has been to explore the extent of bacterial community perturbations in the gut caused by anthropogenic intervention and investigate resulting ramifications on animal fitness and longevity [13,14,15,16,17,18]. Recent investigations have focused on comparing gut microbiota of captive animals and their wild counterparts in order to assess links to the captive lifestyle [14,19,20,21]. Roles of microbiota on host survival upon release have also been examined. Collectively, these studies support monitoring of the microbiota of animals involved in re-introduction and/or translocation projects. Regrettably, the majority of studies fail to include intestinal protozoa, even though emerging evidence suggests that some species persist as asymptomatic colonisers of the intestinal tract [13,22,23,24,25,26,27,28]. Recent studies have showcased that water voles constitute an attractive model for examining these questions [22,23].

The European water vole (*Arvicola amphibius*) is a semi-aquatic rodent that was widespread across Britain in the early 1900s. However, in the past few decades its population has decreased drastically, disappearing from over 89% of previously occupied sites. This has been attributed mainly to habitat destruction and an invasive alien species (IAS), the American mink (*Neovison vison*) [29]. In the UK, attempts are being made to repair fractured populations and re-introduce this mammal into protected wetlands [30,31]. An emerging factor in achieving both of these objectives is the gut microbiota, whose composition in water voles has not been explored. Despite this, previous studies have demonstrated high prevalence and co-occurrence of several eukaryotic microbes in the stool of water voles, hinting at potentially important roles as well [23]. Specifically, the prevalence of protozoan parasites in captive and wild water voles and their associated gut flora remain little explored. The few studies that have examined gut protists in these animals have shown that the most common organism is *Blastocystis*, a microbial eukaryote of questionable pathogenicity.

Herein we investigate the gut microbiota of captive water voles, some of which are involved in re-introductory and breeding schemes. Our annotation and characterisation of the gut microbiota encompassed not only the bacterial component, but also included the protozoan parasites *Cryptosporidium*, *Giardia*, and the questionable pathogen *Blastocystis*. Information on the prokaryotic and eukaryotic components was collectively considered in order to explore their associations in the gut of voles. This study provides the first investigation on association of *Blastocystis* with bacterial communities in the gut of captive water voles. This type of information can assist in re-shaping the strategies for re-introduction programmes of voles into the wild.

## 2. Materials and Methods

### 2.1. Study Site, Animals and Sample Preparation

A conservation park situated in the Southeast, United Kingdom was the subject of this study; Wildwood Trust, Herne Bay, Kent, United Kingdom (51°19′54.1″ N 1°07′10.1″ E) is a wildlife park that houses native British wildlife, housing vertebrate and invertebrate species, that also includes a selection of non-native species that assist with the organisation’s education programme. The park aims to educate the general public on the ecology and status of resident animals in addition to participating in several conservation programmes aiming to ‘re-wild’ Britain. The park is actively involved in breeding, re-introduction and mitigation services for the European water vole (*Arvicola amphibius*) and in recent years has been involved in the release of several individuals. There are over 60 of these rodents currently housed within the park, and their health and breeding status are closely monitored by a licenced veterinarian and keepers. The animals are also monitored for presence of infectious disease agents. During one of these health screenings, *Giardia* was detected in a large number of water voles. Thus, the animals were categorised as either infected or non-infected with *Giardia* and housed accordingly; non-infected and suspected infected individuals were housed in separate enclosures. To minimise risk of transmission, enclosures housing non-infected individuals are cleaned first using separate housekeeping equipment. Following the screening, definitive diagnosis for giardiasis was made by the park veterinarian as follows: faecal samples were collected from three adjacent cages at a time and collated faecal samples were examined for *Giardia* by direct smear. The veterinarian would group collated faecal from three adjacent cages at a time and screen for *Giardia* spp. If they tested positive, all individuals were placed on anti-protozoal treatment with either metronidazole or fenbendazole. Metronidazole treatment lasted 5 days; the dosage was 0.8 mL of a 5 mg/mL injectable solution (approximately 4 mg/vole). Fenbendazole treatments varied in duration; dosage was typically 0.25 mL of a 20% dilution (200 mg/mL original concentration) per 200 g vole. All drug treatments had ended between 10 and 14 days prior to sample collection.

### 2.2. Sample Collection

In this study, a total of 16 water voles housed within the park were sampled between 18 January 2019 and 20 February 2019. A total of 29 faecal samples were collected. Sampling successfully occurred across two collection dates for 12 voles, and a single sample collection took place for four of the voles (R4, Q49, R95 and R34); this was due to no suitable faecal sample for R4 (first collection), Q49 and R95 (second collection), and the death of R34 before the second collection. R13 also died on the morning of the second collection; however, a faecal sample for this vole was obtained before the enclosure was cleaned. Therefore, a total of 12 voles had two successful collections. Samples were collected shortly after enclosures had been cleaned and with the guidance of the park keepers. Only fresh faecal samples were collected. Upon collection, samples were placed in sterile tubes and stored at 4 °C within one hour of collection. At the time of study all voles were considered healthy as stated by a licenced veterinarian and lacked symptoms of gastrointestinal disease.

### 2.3. DNA Extraction, Polymerase Chain Reaction, Cloning

Genomic DNA was extracted directly from approximately 250 mg of the fresh faecal sample using the Microbiome DNA Purification Kit, Purelink (Fisher, California, USA) according to the manufacturer’s instructions. DNA was eluted in 100 μL elution buffer and the working stock stored at −20 °C. Genomic DNA was used for polymerase chain reaction (PCR) with primers targeting gene regions of *Blastocystis*, *Cryptosporidium* and *Giardia* according to previously described protocols [23] (Appendix A Table A1). The purified gel extracts were eluted, of which 1.5 μL was used for cloning with the pGEM-T easy vector system I (Promega, Madison, WI, USA). Between five and ten colonies per transformation were inoculated and grown overnight in 5 mL LB media. The plasmid DNA was extracted using the GeneJet Plasmid Miniprep Kit and clones were confirmed as positive using *Eco*RI (Promega) restriction digestion. Positive clones were sent for sequencing using the T7 and/or SP6 universal primers (Eurofins, Ebersberg Germany).

### 2.4. Amplicon Sequencing of 16S rRNA

Twenty-eight genomic DNA samples were used for microbiome profiling analysis using the Illumina platform (paired-end sequencing). Bacterial taxonomic profiling was carried out by targeting the V1–V3 hypervariable regions of the 16S rRNA gene using the following primers: forward sequence fD2: AGAGTTTGATCATGGCTCAG [32] and reverse sequence S-D-Bact-0008-a-S-20, S-*-Univ-0519-a-A-18: GTATTACCGCGGCTGCTG [33]. All data have been submitted to GenBank under Bioproject number SUB9442672.

### 2.5. Microbiome and Statistical Analysis

Initial bioinformatics analysis was performed using the INVIEW Microbiome Profiling 3.0. Reads with ambiguous bases were removed and chimeric sequences were detected and removed based on the algorithm of UCHIME via the VSEARCH package [34,35]. Where necessary, reads were merged using FLASH software (Baltimore, Maryland) (V2.2.00 http://ccb.jhu.edu/software/FLASH/—access date: February 2020) [36]. Primer and adaptor sequences were removed using Cutadapt [37]. High-quality reads were processed using Minimum Entropy Decomposition (MED) [38,39]. Taxonomy assignment of OTUs was carried using the NCBI sequence database (version 10 October 2019). The most specific taxonomic assignment for each OTU was then transferred from the best-matching reference sequence set and a sequence identity of 70% across a minimum of 80% of representative sequences was the threshold for consideration of a reference sequence (representing a 97% threshold). Further processing of OTUs and taxonomy assignments as part of the INVIEW Microbiome Profiling pipeline was performed using QIIME (Arizona, USA) (version 1.9.1 http://qiime.org/, accessed on 1 February 2020) and OTU abundance normalisation was employed using CopyRighter [40].

All subsequent analyses, including microbial diversity analysis, were performed using the MicrobiomeAnalyst pipeline [41]. Data filtering of OTU data consisted of low count filtering to remove reads with low counts across few samples this was set to a minimum count of four with a prevalence of 20% in samples. Data variance was measured using the inter-quartile range (IQR) and low-variance filtering was implemented to remove features that were close to constant throughout the dataset. Data normalisation was used to facilitate data comparison and to account for unevenness in sampling sparsity and depth; normalisation approaches considered herein included rarefaction to even sequencing depth, data scaling and data transformation.

Microbial diversity was analysed at different taxonomic levels between infected and uninfected voles taking into consideration collection date and protist. Specifically, microbial communities of voles infected with only *Blastocystis*, *Cryptosporidium*, or *Giardia* were compared against uninfected control voles. Moreover, voles infected with more than one protist were also compared against the controls.

Diversity analysis included alpha (within sample) and beta (between samples) measures. Alpha diversity was measured using the Observed species (OS), Chao1 and Shannon indices accounting for OTU richness and evenness. Corresponding statistical significance was determined using the Mann-Whitney U test. The results were displayed using boxplots. Beta diversity was measured using Bray-Curtis Index distance. Corresponding statistical significance was assessed via Permutational Multivariate Analysis of Variance Using Distance Matrices (PERMANOVA). The results were presented as principal coordinates analysis plots (PCoA) to illustrate relationships between the vole microbiomes based on infection category.

To assess the microbial community abundances between different experimental variables, we employed two analyses to identify differentially abundant communities. Classical univariate analysis was used to identify differentially abundant community profiles using Mann-Whitney U (two variables) and Kruskal-Wallis (three variables) analysis based on a single grouping experimental variable. Bioconductor MetagenomeSeq analysis [42], accessed via the Microbiomeanalyst platform, was also used to account for the effects of normalisation and under-sampling of microbial communities. This method was implemented in addition to classical univariate analysis as it is specifically designed to address normalisation and biases in measurements across taxonomic features by way of a zero-inflated Gaussian distribution model to account for the variety in sequencing depth. This method is beneficial, as it aids in the detection of differentially abundant rare taxa.

LEfSE (Linear discriminant analysis Effect Size) was used to perform non-parametric factorial Kruskal-Wallis sum-rank test to identify which community abundance features were significantly different with regard to the experimental factor and most likely to explain differences between experimental variables.

## 3. Results

### 3.1. Occurrence of Intestinal Protists

Of the 29 faecal samples collected from 16 voles, DNA was successfully extracted from 28 of these and was screened for *Cryptosporidium*, *Giardia* and *Blastocystis* (Table 1). *Blastocystis* was detected in 7/16 voles (44%) and in 9/28 samples (32%). A total of 39 clones were sent for sequencing and 16/39 were positive (41%). Of the 28 successfully extracted faecal samples, 5/28 and 4/16 (25%) voles were sequence positive for *Giardia* A total of 95 clones were sent for sequencing, of which 11/95 (12%) were sequence positive. A total of 5/28 (18%) samples and 5/16 voles (31%) were sequence positive for *Cryptosporidium* and generated 11/68 positive clones (16%).

### 3.2. Characterisation of Bacterial Communities in the Stool

A total of 2,469,175 reads were obtained from 16S rRNA amplicon sequencing. After quality filtering and processing, the total read count measured at 1,509,628, with an average of 53,915 reads per sample. The maximum and minimum counts per sample were 76,169 and 34,050, respectively. The final operational taxonomic unit (OTU) number was 778. Low count filtering was applied, with a minimum count of four reads at a 20% prevalence across the samples. Data normalisation was used to account for the large variability of total read counts between samples. The library was not rarefied, in order to reduce loss of possibly significant data from high sequence counts due to the relatively small difference in library sizes (<10×). Variance filtering screened out features that were close to constant and was measured using the inter-quartile range, which was set to a 10% threshold. A total of 162 low-abundance features were removed based on low read count and 20 low-variance features were removed based on the inter-quartile range; 171 features remained. The data were scaled via total sum scaling to address uneven sequencing depth.

### 3.3. Taxonomic Composition Diversity and Community Profiling

**Figure 1:**
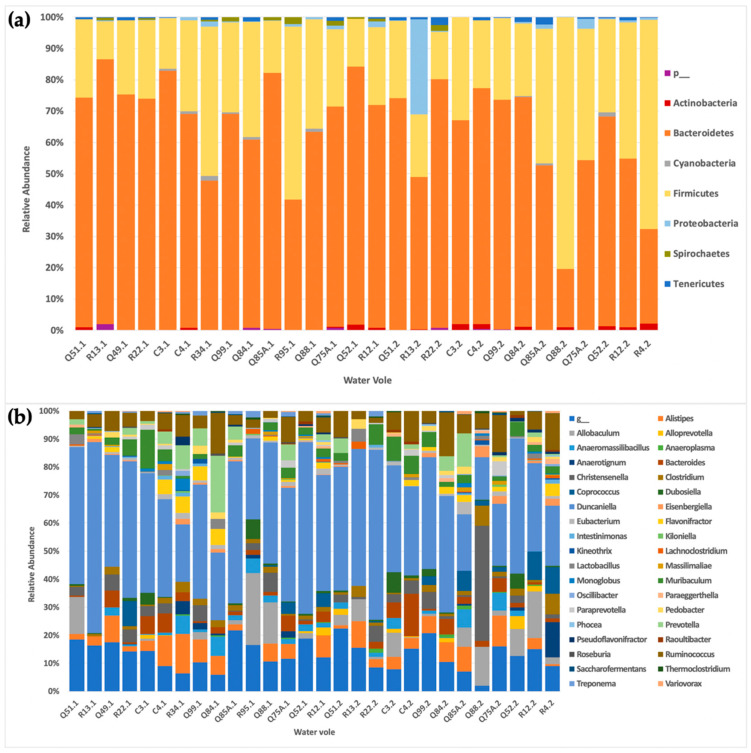
Relative abundance of OTUs across the sample population. (**a**) Relative taxa abundance of the sampled water voles at the phylum level. Across the majority of voles, *Bacteroidetes* (orange) dominate. These are followed by the *Firmicutes* (yellow), which collectively account for over 90% of the out abundance at the phylum level. R13.1 shows a significant increase in *Proteobacteria* (light blue). “p-” signifies all mergoutOTU phylum data that have a relative abundance that is below 1% across samples. (**b**) Relative taxa abundance of the sampled water voles at the genus level. Across the majority of voles, the relative abundance of OTUs at the genus level is consistent. One notable observed difference is the relative increase in *Christensenella* (dark grey) in Q88.1. “g-” signifies all merged OTU genus data that have a relative abundance that is below 1% across samples.

There are few overall observable differences in the taxonomic composition of the samples in the present study. OTUs spanned seven phyla and all but one sample was dominated by *Bacteroidetes* (63% relative abundance across all samples) followed by *Firmicutes* (31% relative abundance across all samples), with the exception of four samples, which were dominated by *Firmicutes*. *Actinobacteria*, *Cyanobacteria*, *Proteobacteria* and *Tenericutes* were observed. Excluding *Bacteroidetes* and *Firmicutes*, the remaining phyla accounted for less than 10% of overall abundance. Vole R13 notably varied at the phylum level and was dominated by *Proteobacteria* at a relative abundance of 32% compared with the 1% relative abundance average for the rest of the voles in the second time point (Figure 1).

Overall, less than 2% of reads were not assigned at the phylum level. At the family level, the number of unassigned OTUs was 50%, while at the genus level this was over 60%. At the genus level, approximately 38% of the remaining (relative) abundance was composed of members of *Duncaniella*, followed by *Ruminococcus* (7%), *Alistipes* (5%), *Allobaculum* (5%), *Muribaculum* (4%), *Christensenella* (4%), *Prevotella* (3%), *Bacteroides* (3%), *Clostridium* (2%), *Coprococcus* (2%), *Anaeromassilibacillus* (2%), *Flavonifractor* (2%), *Alloprevotella* (1%), *Anaerotignum* (1%), *Dubosiella* (1%), *Eisenbergiella* (1%), *Eubacterium* (1%), *Lactobacillus* (1%), *Prarprevotella* (1%), *Pedobacter* (1%) (Figure 1).

Abundances did not differ in terms of sampling time points, with the following exceptions: at the phylum level, the relative abundance of *Proteobacteria* in vole R13 in the first collection (R13.1; Figure 1a) was <1%, while this increased to approximately 32% in the second collection (R13.2; Figure 1a). At the genus level, the vole Q88 had an average abundance of *Christensenella* of 7% in the first collection (Q88.1; Figure 1b), yet its abundance was greatly increased to over 40% in the second collection (Q88.2; Figure 1b).

Relative abundances of OTUs between *B+*, which were negative for other protozoa (n = 5), were also compared against the *B*− samples, *Cryptosporidium* and *Giardia* (n = 14) (Table A2). To minimise the impact of co-parasitism, results herein for *Blastocystis* are based on comparisons made between these two groups.

**Figure 2:**
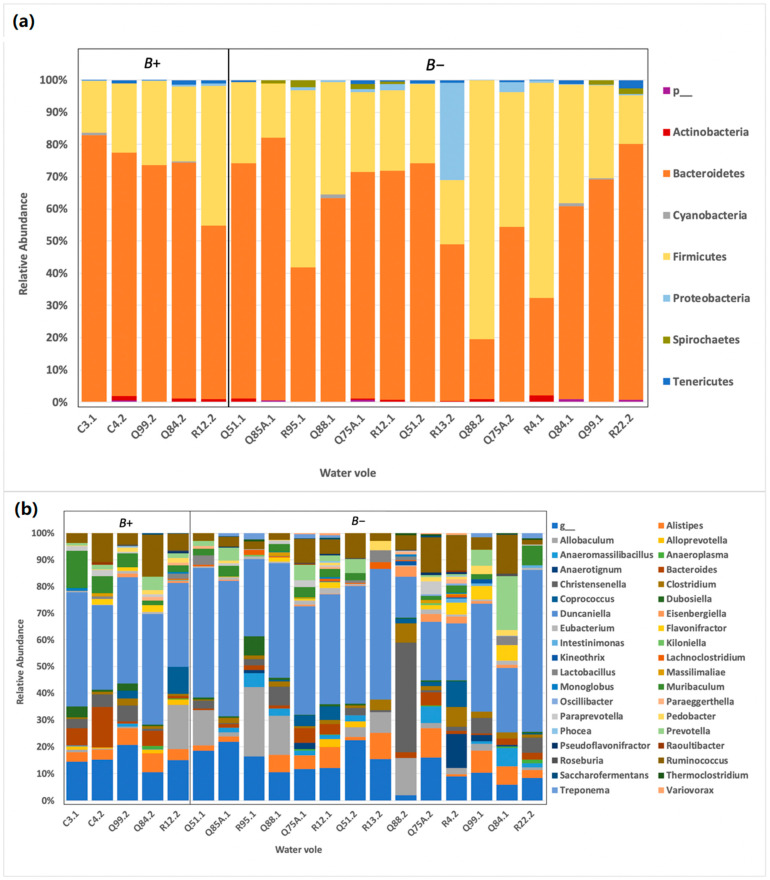
Relative abundance of OTUs separating *Blastocystis* and negative samples. (**a**) Relative taxa abundance of *Blastocystis* positive voles *B+* and *Blastocystis* negative voles *B−* at the phylum level. Between the two groups, the relative abundance of OTUs at the phylum level are consistent. (**b**) Relative taxa abundance of *Blastocystis* positive voles *B+* and *Blastocystis* negative voles *B−* at Table 1 across all samples, where g_ signifies all merged OTU genus data that have a relative abundance that is below 1% across all samples.

The relative abundances for *B+* and *B−* voles at the phylum level (Figure 2a) and the genus level (Figure 2b) are displayed below, using the selected data detailed in Table A1.

We also performed a treated- versus untreated-animals analysis, but no significant differences were found (data not shown), either due to the small sample size (Metronidazole-treated animals = 3; Fenbendazole-treated animals = 5) or a result of the amount of time elapsed following treatment. This was the result of the treatment regimens implemented by the veterinary practice.

### 3.4. Microbial Diversity Measures

**Figure 3:**
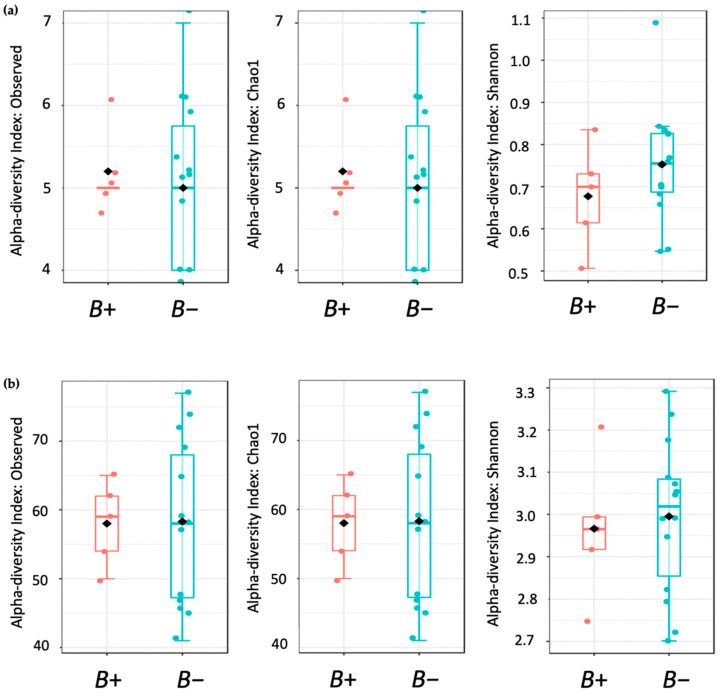
Boxplots showing alpha diversity (Observed, Chao1, and Shannon indices). Blue plots represent the negative (*B−*) samples and plots in red represent positive (*B*+) voles. (**a**) Alpha diversity boxplots at the phylum level; (**b**) results at the genus level.

Alpha diversity was quantified using three methods: Observed species, Chao1, and Shannon indices. The Shapiro-Wilk test for normality classified the data as non-normally distributed; thus, non-parametric tests were used for all statistical analyses. For each result, between-group variations were measured using Mann-Whitney U test. Overall, no significant difference in OTU richness was observed between *B+* and *B−* voles (Table A3, Figure 3).

**Figure A1:**
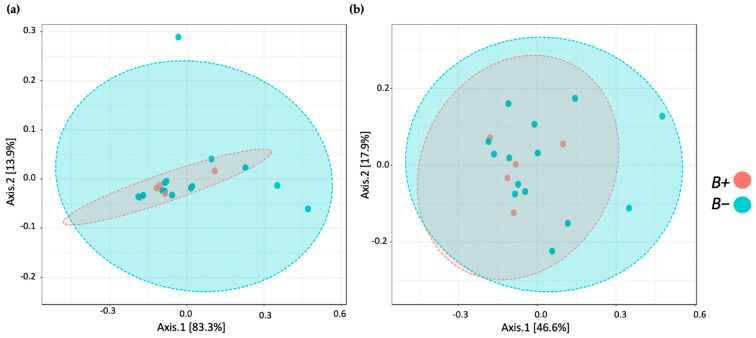
Principal Coordinate Analysis (PCoA) plots of the beta diversity of *B*+ voles (red) and *B*- voles (blue) based on Bray-Curtis dissimilarity index (**a**) shows the Beta-diversity PCoA plot at the phylum level and (**b**) shows the Beta-diversity PCoA at the genus level.

Beta diversity measures were implemented using the Bray-Curtis dissimilarity index. This was accompanied by a PERMANOVA test to determine if centroids differed between variables of interest. Analysis was visualised with 2D ordination plots based on principal coordinate analysis (PCoA) (Table A4, Figure A1). The results showed no significant difference between the microbial communities of positive voles against negative voles (*p* < 0.05).

### 3.5. Microbial Community Comparison and Classification

**Figure 4:**
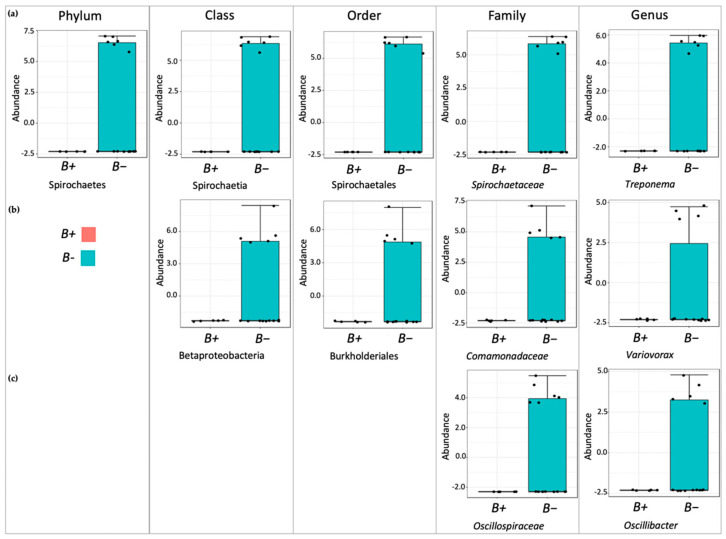
Boxplots showing the log transformed counts of distinguished OTUs identified by MetagenomeSeq analysis (*p* < 0.05). This includes multiple taxa belonging to the same lineage: (**a**) OTUs belonging to the *Spirochaetes* lineage; (**b**) OTUs belonging to the *Betaproteobacteria* lineage; (**c**) OTUs belonging to the *Oscillospiraceae* lineage. Plots in blue represent the data from *B−* voles, red plots are from *B*+ voles.

**Figure A2:**
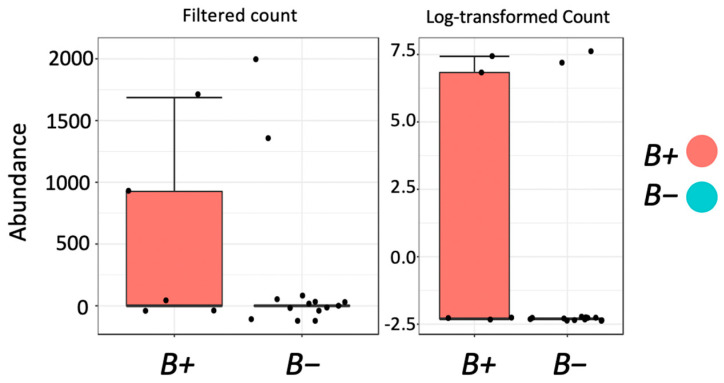
Box-plots showing the filtered count and log transformed counts from MetagenomeSeq data of the genus *Anaerocella.* This was the only bacterial taxon positively associated with *Blastocystis* colonisation in the study cohort (*p <* 0.05). Red plots reflect *B*+ data, and blue plots represent *B*- data.

To calculate the microbial community abundance, we used classical univariate analysis and MetagenomeSeq. Classical univariate analysis revealed no significant differences between *B*+ and *B−* voles (*p* < 0.05). MetagenomeSeq identified a total of 19 significant results when *B*+ was compared against *B−* (Table A5) (*p* < 0.05) and community abundance across different OTUs, as displayed in Table A6. Members of the *Spirochaetes* lineage were most notably negatively associated with the presence of *Blastocystis,* where a significant decrease was observed from the phylum to the genus *Treponema*. Members of the *Betaproteobacteria* lineage were also decreased in the presence of *Blastocystis,* and included the genus *Variovorax*. A total of nine genera decreased in the presence of *Blastocystis*, with *Anaerocella* being the only taxa to increase. Figure 4 displays boxplots for the log transformed count of significant OTUs visually representing the data presented in Table A6, including bacteria belonging to the same taxonomic lineages. All identified OTUs were significantly decreased in *B+* voles, only the genus *Anaerocella* was significantly increased (Figure A2).

Lastly, Linear Discriminant Analysis (LDA) Effect Size (LEfSe) was implemented to investigate community comparisons. This method determines which OTU was most likely to explain the differences between classes by using standard statistical significance tests with additional tests to consider biological consistency and effect relevance. LEfSe using FDR-adjusted data (*p* value cut-off = 0.1) demonstrated that no significant taxa were observed when *B*+ voles were compared against *B−* voles.

## 4. Discussion

This pilot study provides the first investigation exploring the association of *Blastocystis* with bacterial communities in the gut of captive water voles (*Arvicola amphibius*) presenting no gastrointestinal symptoms at the time of collection. Twenty-nine samples were collected over two time points and the microbiome of twenty-eight of these was characterised. Of these, 44% were *Blastocystis* positive and were subsequently subtyped, and associations with bacterial microbiome were examined for the first time in this rodent.

Several studies on other rodents do exist, including those of the subfamily *Arvicolinae* [43,44,45]. Generally, the core *Arvicolinae* microbiota was made up predominantly of *Firmicutes* and *Bacteroidetes*, and this was the case here, with the majority of the water voles reporting *Bacteroidetes* and *Firmicutes* as the two dominant phyla, reflecting results across other mammalian studies [46]. Specifically, the abundance of *Bacteroidetes* was higher (65%) in comparison to that of *Firmicutes*. Previous vole-based studies of captive and wild-captured cohorts have shown similar abundances [43]. In general, studies in humans and rodents have shown that *Bacteroidetes*-driven microbiota may be the result of low fat/high fibre diet [47,48,49,50]. Nonetheless, rats showed a less than 10% abundance of *Bacteroidetes*, possibly reflecting their omnivorous nature [51]. The water voles in this study were fed a diet rich in fibrous material including fruits, legumes, willow leaves and bark, which likely accounts towards the high abundance of *Bacteroidetes* [45]. Besides the two dominant phyla, the bacterial communities of the water voles diversified further at the lower taxonomic levels, in accordance with numerous microbiome-based studies [52,53]. More research investigating composition of the bacterial community and abundance of individual taxa in a wide range of vole species will help establish the gut microbiome makeup of these small rodents.

An exception to the above was noted in one water vole, which displayed a markedly different abundance profile from the rest during the second collection. Specifically, this water vole (R13) had a drastic change in the relative abundance of *Proteobacteria* between collections, with the value increasing from <1% in the first collection to over 30% in just one month. This alteration was also accompanied by a decrease in the relative abundance of *Bacteroidetes* from 85% to 49% between collections. The water vole that died from unknown causes on the morning of the second collection had a high abundance of *Proteobacteria*, which is often associated with dysbiosis in animal studies including humans, where it has been linked to both intestinal related diseases, such as Crohn’s disease, as well as extra-intestinal disease possibly indicating a disruption of enteric homeostasis [54,55,56,57,58]. This implies a use for *Proteobacteria* as a biomarker for intestinal dysbiosis in captive water voles.

In recent years, hypotheses regarding the association of opportunistic protists with distinct microbial profiles have been brought forth [59,60,61,62,63]. Among those, the most-studied protist is *Blastocystis*, which has been suggested to be an “ecosystem engineer” [64]. Presence of *Blastocystis* has been associated with an increase in overall bacterial diversity and richness. A negative association with *Bacteroides* and presence of *Blastocystis* has been consistently found across human studies [64,65,66,67]. Positive associations with *Roseburia* and *Faecalibacterium*, which are often associated with eubiosis, have also been noted [68,69]. Presence of this protist has also been linked to a decrease in *Hymenolepis nana*, which has been associated with alterations in the microbiota [70,71], these results led to the hypothesis that *Blastocystis* is part of the healthy intestinal microbiome in humans. Contrary to this, a single study focusing on chimpanzees demonstrated that bearing *Blastocystis* was associated with decreased microbial richness and decline in the ‘protective’ species *Faecalibacterium prausnitzii* and increase in *Enterobacteriaceae*, a marker of poor intestinal health in humans [62]. Herein, contrary to the human studies, we found no significant differences between the microbial profiles of water voles with and without *Blastocystis*. A possible explanation could be that the presence of *Blastocystis* in water voles might not have the same associations as those observed in humans. Other possible hypotheses could be the homogenisation of bacterial taxa due to captivity and/or disturbances due to drug administration. Due to the small size (16 individuals, <30 samples), we cannot at this point draw a definitive conclusion.

Although the overall microbial community richness was similar in water voles with and without *Blastocystis*, closer inspection of community comparisons indicated that reductions in *Treponema* and *Kineothrix* were strongly associated with *Blastocystis* presence. *Treponema* has been associated with degradation of plant materials in the rumen [72]. In support of this, *Treponema* has been found in significantly higher abundances in the gut of humans living in rural areas and eating fibre-rich diets, while it is typically absent in urbanites consuming fibre-poor diets [73,74]. The bacterium has also been associated with the vole microbiota, where it likely has similar roles [44]. Nonetheless, it is worth noting that other common degraders, such as *Prevotella*, *Ruminococcus* and *Oscillospira*, were not significantly decreased in *Blastocystis* carriers herein. This suggests that the observed decline in *Treponema* likely does not impair the ability of the water vole to degrade plant materials. *Kineothrix* was also significantly decreased in positive voles, this bacterium produces butyrate, a metabolite that serves as energy source of enterocytes and has notable anti-inflammatory and immunomodulatory properties [75]. Furthermore, the *Firmicutes* genera *Thermoclostridium, Anaeromassilibacillus* and *Anaerotignum* were also decreased in positive water voles; however, most of these have uncharacterised roles in the murine microbiota. Nonetheless, *Firmicutes* are generally associated with fermentation of dietary fibre and production of short-chain fatty acids [76]. Overall, the observed significant reductions of specific bacterial taxa may represent the beginnings of a disturbed gut, which is a hallmark of the transition from free-living to a more confined lifestyle. Nonetheless, definitive conclusions cannot be drawn, as there was redundancy of function in the gut, which may be enhanced by the presence of *Blastocystis*, as the organism has been associated with increased species richness in the gut [67,77,78,79]. Alternatively, the observed shifts might be associated with an as-yet-unidentified factor other than *Blastocystis*.

Several of the water voles were also colonised with either *Giardia* or *Cryptosporidium* and in several cases with more than just one of these microbial eukaryotes. Notably, the water voles also received the antiprotozoal drugs fenbendazole and metronidazole against *Giardia* and treatments had ended by the time of sampling. Despite this, there were no differences in either alpha or beta diversities in any of these groups. This, along with the absence of diarrhoea or other GI symptoms, suggests that the water voles might be carriers of these parasites.

## 5. Conclusions

In conclusion, these results provide an insight into the prevalence of *Blastocystis* and its association with bacterial communities present in the gut of captive water voles. The apparent lack of symptoms in the cohort and lack of overall shift in community richness and diversity of positive voles indicates that *Blastocystis* may not be associated with a detrimental effect on the gut microbiota. One could also raise questions regarding the necessity of antiprotozoal treatments in asymptomatic animals. Clearly, anthropogenic-focused microbiome studies do not reflect those of animals. Therefore, further investigations into the presence of *Blastocystis* and associated microbial profiles across a range of host taxa in captivity and wild populations will hopefully shed light on the roles of protozoal colonisation and resulting impacts this may pose for conservation efforts.

## Figures and Tables

**Table 1:** Protozoa screening results from the study cohort. Screened parasites included *Blastocystis,* which was the main focus of this study, in addition to *Cryptosporidium* sp. and *Giardia intestinalis*. *G. intestinalis* was being monitored in the vole population prior to this study, and several of the voles were undergoing treatment for *Giardia* infection.

Sample No.	Vole ID	Col. Date	Microbiome Profile ID	Drug Treatment Prior to Sampling	Vet Diagnosis for *Giardia*	Screening Result			Sample No.	Vole ID	Col. Date	Microbiome Profile ID	Drug Treatment Prior to Sampling	Vet Diagnosis for *Giardia*	Screening Result		
						*Giardia*	*Cryptosporidium*	*Blastocystis*							*Giardia*	*Cryptosporidium*	*Blastocystis*
**1**	C3	18.01.19	C3.1	Fenbendazole	+			4	**1**	C3	20.02.19	C3.2	None	+	+		4
**1**	C4	18.01.19	C4.1	Fenbendazole	+		+	4	**1**	C4	20.02.19	C4.2	None	+			4
**1**	Q84	18.01.19	Q84.1	Metronidazole	+				**1**	Q84	20.02.19	Q84.2	Fenbendazole	+			1
**1**	R22	18.01.19	R22.1	Fenbendazole	+		+	4	**1**	R22	20.02.19	R22.2	None	+			
**1**	R34	18.01.19	R34.1	Metronidazole	+		+										
**1**	Q99	18.01.19	Q99.1	Metronidazole	+				**1**	Q99	20.02.19	Q99.2	Fenbendazole	+			4, *B.lapemi*
**1**	Q49	18.01.19	Q49.1	None	+	+	+										
**1**	Q51	18.01.19	Q51.1	None	+				**1**	Q51	20.02.19	Q51.2	None	+			
**1**	R13	18.01.19	R13.1	None	+		+		**1**	R13	20.02.19	R13.2	None	+			
**1**	Q52	18.01.19	Q52.1	None	-	+			**1**	Q52	20.02.19	Q52.2	None	-	+		*B. lapemi*
**1**	Q75A	18.01.19	Q75A.1	None	-				**1**	Q75A	20.02.19	Q75A.2	None	-			
**1**	Q85A	18.01.19	Q85A.1	None	-				**1**	Q85A	20.02.19	Q85A.2	None	-	+		
**1**	Q88	18.01.19	Q88.1	None	-				**1**	Q88	20.02.19	Q88.2	None	-			
**1**	R12	18.01.19	R12.1	None	-				**1**	R12	20.02.19	R12.2	None	-			1, 4
**1**	R95	18.01.19	R95.1	None	-												
									**1**	R4	20.02.19	R4.2	None	-			

## Data Availability

All data have been submitted to GenBank under Bioproject number SUB9442672.

## Appendix A

**Table A1:** Summary of PCR primers used in this study.

Target Organism	Gene	Primer Pair	Reference
*Blastocystis*	18s rRNA	RD3 / RD5	[80,81]
*Blastocystis*	18s rRNA	RD5F / BhRDr	
*Cryptosporidium*	18s rRNA	CRY F1 / CRY R1	[82]
*Cryptosporidium*	18s rRNA	RDY F2 / CRY R2	
*Giardia*	*gdh*	GDHeF / GDHiF	[83]
*Giardia*	*gdh*	GDHeF / GDHiR	

**Table A2:** Summary of the voles that will be included in subsequent analysis for *Blastocystis*-related investigation. Previous drug treatment (ending 10+ days prior to collection) was also recorded.

Water Vole ID	Collection Date	*Blastocystis* Positive	Prior Drug Treatment
C3	18.01.19	Yes	Yes
C4	20.02.19	Yes	No
R12	20.02.19	Yes	No
Q99	20.02.19	Yes	Yes
Q84	20.02.19	Yes	Yes
Q51	18.01.19	No	No
Q51	20.02.19	No	No
Q75A	18.01.19	No	No
Q75A	20.02.19	No	No
R22	20.02.19	No	No
Q85A	18.01.19	No	No
Q88	18.01.19	No	No
Q88	20.02.19	No	No
R12	18.01.19	No	No
R13	20.02.19	No	No
R4	20.02.19	No	No
R95	18.01.19	No	No
Q84	18.01.19	No	Yes
Q99	18.01.19	No	Yes

**Table A3:** Alpha diversity results for *Blastocystis* positive (*B+*) voles compared with negative (*B-*) voles. Diversity was measured using three methods: Observed, Chao1 and Shannon indices at each taxonomic level. No statistically significant results were identified (*p* < 0.05).

Experimental Factor	Taxonomic Level	Diversity Measure	*p*-Value	Mann-Whitney Statistic
***Blastocystis***	Phylum	Observed	0.55111	41.5
		Chao1	0.55111	41.5
		Shannon	0.3913	25
	Class	Observed	0.34373	45.5
		Chao1	0.34373	45.5
		Shannon	0.2193	21
	Order	Observed	0.45552	43.5
		Chao1	0.45552	43.5
		Shannon	0.2193	21
	Family	Observed	0.67615	40
		Chao1	0.67615	40
		Shannon	0.34262	24
	Genus	Observed	0.88941	37
		Chao1	0.88941	37
		Shannon	0.68679	30

**Table A4:** Beta diversity results for *Blastocystis* positive (*B*+) voles compared with negative (*B*-) voles. Diversity was measured using Bray-Curtis dissimilarity index with Permutational Multivariate Analysis of Variance Using Distance Matrices (PERMANOVA). No significant results were identified (*p* < 0.05).

Experimental Factor	Taxonomic Level	F-Value	R-Squared Vale	*p*-Value
***Blastocystis***	Phylum	1.1208	0.061854	<0.311
	Class	0.82066	0.046051	<0.44
	Order	0.81818	0.045918	<0.444
	Family	0.72235	0.040759	<0.597
	Genus	0.82372	0.046215	<0.519

**Table A5:** Significant OTUs identified by MetagenomeSeq as differentially abundant between *B*+ and *B*- samples (*p* < 0.05). A total of 19 significant results were identified here, the observed difference in community abundance summarises the observed change between *B*+ and *B*- for each OUT.

Experimental Factor	OTU	Name	Observed Difference in Community Abundance	*p*-Value	FDR
***Blastocystis***	Phylum	Spirochaetes	Decrease in infected	5.43 × 10^−9^	4.35 × 10^−8^
	Class	Spirochaetia	Decrease in infected	1.15 × 10^−9^	1.49 × 10^−8^
	Class	Betaproteobacteria	Decrease in infected	3.48 × 10^−7^	2.26 × 10^−6^
	Class	Epsilonproteobacteria	Decrease in infected	4.43 × 10^−5^	1.92 × 10^−4^
	Order	Spirochaetales	Decrease in infected	3.86 × 10^−8^	5.78 × 10^−7^
	Order	Burkholderiales	Decrease in infected	1.37 × 10^−6^	1.03 × 10^−5^
	Family	*Spirochaetaceae*	Decrease in infected	1.26 × 10^−8^	3.03 × 10^−7^
	Family	*Oscillospiraceae*	Decrease in infected	8.01 × 10^−7^	9.61 × 10^−6^
	Family	*Comamonadaceae*	Decrease in infected	4.38 × 10^−6^	3.50 × 10^−5^
	Genus	*Treponema*	Decrease in infected	1.90 × 10^−8^	8.35 × 10^−7^
	Genus	*Variovorax*	Decrease in infected	2.17 × 10^−6^	4.77 × 10^−5^
	Genus	*Kineothrix*	Decrease in infected	5.33 × 10^−5^	6.99 × 10^−4^
	Genus	*Oscillibacter*	Decrease in infected	6.36 × 10^−5^	6.99 × 10^−4^
	Genus	*Robinsoniella*	Decrease in infected	4.30 × 10^−4^	3.79 × 10^−3^
	Genus	*Thermoclostridium*	Decrease in infected	1.40 × 10^−3^	1.03 × 10^−2^
	Genus	*Kiloniella*	Decrease in infected	3.06 × 10^−3^	1.92 × 10^−2^
	Genus	*Anaeromassilibacillus*	Decrease in infected	5.77 × 10^−3^	2.99 × 10^−2^
	Genus	*Anaerotignum*	Decrease in infected	6.11 × 10^−3^	2.99 × 10^−2^
	Genus	*Anaerocella*	Increase in infected	7.99 × 10^−3^	3.52 × 10^−2^

**Table A6:** Taxa table showing the relationships between identified OTUs via MetagenomeSeq. The observed change in OTU abundance in *B*+ voles compared with *B*- voles is summarised in the ‘Result’ column.

Phylum	Class	Order	Family	Genus	Result
Spirochaetes	Spirochaetia	Spirochaetales	*Spirochaetaceae*	*Treponema*	Decrease
	Betaproteobacteria	Burkholderiales	*Comamonadaceae*	*Variovorax*	Decrease
	Epsilonproteobacteria				Decrease
			*Oscillospiraceae*	*Oscillibacter*	Decrease
				*Kineothrix*	Decrease
				*Robinsoniella*	Decrease
				*Thermoclostridium*	Decrease
				*Kiloniella*	Decrease
				*Anaeromassilibacillus*	Decrease
				*Anaerotignum*	Decrease
				*Anaerocella*	Increase
